# Conservation of core complex subunits shaped the structure and function of photosystem I in the secondary endosymbiont alga *Nannochloropsis gaditana*

**DOI:** 10.1111/nph.14156

**Published:** 2016-09-13

**Authors:** Alessandro Alboresi, Clotilde Le Quiniou, Sathish K. N. Yadav, Martin Scholz, Andrea Meneghesso, Caterina Gerotto, Diana Simionato, Michael Hippler, Egbert J. Boekema, Roberta Croce, Tomas Morosinotto

**Affiliations:** ^1^ Dipartimento di BiologiaUniversità di PadovaVia U. Bassi 58/B35121PadovaItaly; ^2^ Department of Physics and Astronomy and Institute for Lasers, Life and BiophotonicsFaculty of SciencesVU University AmsterdamDe Boelelaan 10811081 HVAmsterdamthe Netherlands; ^3^ Electron Microscopy GroupGroningen Biomolecular Sciences and Biotechnology InstituteUniversity of GroningenNijenborgh 79747 AGGroningenthe Netherlands; ^4^ Institute of Plant Biology and BiotechnologyUniversity of MünsterMünster48143Germany

**Keywords:** electron microscopy, Heterokonta, Light‐harvesting complex, *Nannochloropsis*, photosynthesis, photosynthetic apparatus, photosystem

## Abstract

- Photosystem I (PSI) is a pigment protein complex catalyzing the light‐driven electron transport from plastocyanin to ferredoxin in oxygenic photosynthetic organisms. Several PSI subunits are highly conserved in cyanobacteria, algae and plants, whereas others are distributed differentially in the various organisms.
- Here we characterized the structural and functional properties of PSI purified from the heterokont alga *Nannochloropsis gaditana*, showing that it is organized as a supercomplex including a core complex and an outer antenna, as in plants and other eukaryotic algae.
- Differently from all known organisms, the *N. gaditana* PSI supercomplex contains five peripheral antenna proteins, identified by proteome analysis as type‐R light‐harvesting complexes (LHCr4‐8). Two antenna subunits are bound in a conserved position, as in PSI in plants, whereas three additional antennae are associated with the core on the other side. This peculiar antenna association correlates with the presence of PsaF/J and the absence of PsaH, G and K in the *N. gaditana* genome and proteome.
- Excitation energy transfer in the supercomplex is highly efficient, leading to a very high trapping efficiency as observed in all other PSI eukaryotes, showing that although the supramolecular organization of PSI changed during evolution, fundamental functional properties such as trapping efficiency were maintained.

## Introduction

In organisms performing oxygenic photosynthesis, Photosystem I (PSI) is responsible for the light‐driven electron transport from plastocyanin/cytochrome 6 to ferredoxin (Raven *et al*., 1999; Nelson & Yocum, 2006; Croce & van Amerongen, 2013; Bernal‐Bayard *et al*., 2015). In eukaryotes, PSI is organized as a supercomplex composed of two moieties, the core and the peripheral antenna system. The PSI core is composed of 12–14 subunits (Busch & Hippler, 2011). PsaA and PsaB bind the reaction center P700 as well as most of the cofactors involved in electron transport and *c*. 100 chlorophylls (Chls) active in light harvesting (Jordan *et al*., 2001; Qin *et al*., 2015). PsaA and PsaB are highly conserved in cyanobacteria, algae and plants (Allen *et al*., 2011). Other core subunits are instead differently distributed in various phylogenetic groups. For instance PsaG and PsaH are only found in plants and green algae (Vanselow *et al*., 2009; Busch & Hippler, 2011) where they serve as docking site for the association of the peripheral antenna (Lunde *et al*., 2000; Ben‐Shem *et al*., 2003).

The peripheral antenna is composed of light‐harvesting complexes (LHC) whose sequences vary in different organisms: LHCa1–6 are present in plants (Jansson, 1999), LHCa1–9 are present in the green alga *Chlamydomonas reinhardtii* (Mozzo *et al*., 2010), whereas LHCR proteins are believed to comprise the peripheral antenna in red algae and diatoms (Busch *et al*., 2010; Busch & Hippler, 2011; Thangaraj *et al*., 2011). The number of LHC associated with PSI is also variable: four subunits are associated with the PSI core in higher plants*,* in the moss *Physcomitrella patens*, and in some red algae and diatoms (Ben‐Shem *et al*., 2003; Veith & Büchel, 2007; Busch *et al*., 2010, 2013). Differently, nine antenna subunits are found associated with PSI in other red algae (Gardian *et al*., 2007; Thangaraj *et al*., 2011) as in the green alga *C. reinhardtii* (Drop *et al*., 2011).

Finally, the oligomeric state of PSI also varies in different organisms. Cyanobacterial PSI often has been reported to be a trimer (Boekema *et al*., 1987; Jordan *et al*., 2001), although PSI tetramers have been identified in a growing number of species (Watanabe *et al*., 2011, 2014; Li *et al*., 2014). However, PSI was found to be monomeric in all eukaryotes analyzed so far, including plants (Ben‐Shem *et al*., 2003; Kouril *et al*., 2005a), diatoms (Veith & Büchel, 2007; Ikeda *et al*., 2013), green and red algae (Gardian *et al*., 2007; Drop *et al*., 2011).

A peculiar feature of PSI is the presence of Chls absorbing at energy lower than the primary electron donor P700, called red forms (Croce & van Amerongen, 2013). Although these far red‐absorbing Chls account only for a small fraction of the total absorption, they have a strong influence in excitation energy transfer and trapping, slowing down the trapping time as they introduce uphill steps in the energy transfer process (Gobets *et al*., 2001; Jennings *et al*., 2003; Engelmann *et al*., 2006; Wientjes *et al*., 2011b). The presence of low energy absorbing Chls is ubiquitous in PSI, but their energy appears to be highly species‐dependent (Gobets & van Grondelle, 2001; Croce & van Amerongen, 2013). In plants, most red forms are associated with the outer antenna complexes and in particular with LHCA3 and LHCA4 (Schmid *et al*., 1997; Castelletti *et al*., 2003), although the core also contains low energy forms (Croce *et al*., 1998; Gobets & van Grondelle, 2001).

In the present work, we investigated the structural and functional properties of the Photosystem I supercomplex (PSI‐LHC) of the eustigmatophycea *Nannochloropsis gaditana*. This microalga belongs to the phylum Heterokonta, which also includes diatoms and brown algae (Cavalier‐Smith, 2004; Riisberg *et al*., 2009), that originated from a secondary endosymbiotic event where an eukaryotic host cell engulfed a red alga (Archibald & Keeling, 2002). In the last few years, species belonging to the *Nannochloropsis* genus have gained increased attention not only for their evolutionary position, but also for their ability to accumulate a large amount of lipids (Rodolfi *et al*., 2009; Bondioli *et al*., 2012; Simionato *et al*., 2013). The *N. gaditana* photosynthetic apparatus presents distinct features with respect to other algae such as the presence of only Chl*a* and an atypical carotenoid composition with violaxanthin and vaucheriaxanthin esters as the most abundant xanthophylls (Sukenik *et al*., 1992, 2000; Basso *et al*., 2014). A deeper characterization of this organism thus contributes to a better understanding of the variability of photosynthetic organisms in an evolutionary context.

## Materials and Methods

### Cell growth and thylakoid isolation

*Nannochloropsis gaditana* from the Culture Collection of Algae and Protozoa (CCAP), strain 849/5, was grown in sterile F/2 medium using 32 g l^−1^ sea salts (Sigma‐Aldrich), 40 mM TRIS‐HCl (pH 8) and Guillard's (F/2) marine water enrichment solution (Sigma‐Aldrich). Cells were grown with 100 μmol of photons m^−2^ s^−1^ of illumination using fluorescent light tubes and air enriched with 5% CO_2_. The temperature was set at 22 ± 1°C. Thylakoid membranes were isolated as described previously (Basso *et al*., 2014).

### Thylakoid solubilization and PSI isolation

Thylakoid membranes (corresponding to 500 μg of Chl) were solubilized with 0.6% α‐DM or 1% β‐DM as described in (Basso *et al*., 2014) and then fractionated by ultracentrifugation in a 0.1–1 M sucrose gradient containing 0.06% α‐DM and 10 mM HEPES (pH 7.5) (280 000 ***g***, 18 h, 4°C). Fractions of the sucrose gradient were then harvested with a syringe. PSI samples isolated after α‐DM or β‐DM solubilization were named PSI‐LHC_α_ and PSI‐LHC_β_, respectively.

### Electron microscopy and image analysis

Four microliters of purified sample was absorbed onto glow discharged carbon‐coated grids and subsequently stained with 2% uranyl acetate for contrast. Imaging was performed on a Tecnai T20 equipped with a LaB6 tip operating at 200 kV. The ‘GRACE’ system for semi‐automated specimen selection and data acquisition (Oostergetel *et al*., 1998) was used to record 2048 × 2048 pixel images at ×133 000 magnifications using a Gatan 4000 SP 4K slow‐scan CCD camera with a pixel size of 0.224 nm. Three hundred thousand particles were picked from 16 000 raw images and single particles were analyzed with xmipp software (including alignments, statistical analysis and classification, as in Scheres *et al*., 2008) and relion software (Scheres & Chen, 2012). The best of the class members were taken for the final class‐sums.

### Sequence analysis

*Emiliania huxleyi*,* Phaeodactylum tricornutum*,* Thalassiosira pseudonana*,* Ectocarpus silicolosus*,* N. gaditana*,* Chlamydomonas reinhardtii*,* Physcomitrella patens*,* Arabidopsis thaliana*,* Oryza sativa*,* Cyanidioschyzon merolae*,* Galdieria sulphuraria* genome databases were accessed online via the National Center for Biotechnology Information (NCBI) portal using TBlastN and BlastP. Additional data were collected from http://bioinformatics.psb.ugent.be/genomes/view/Ectocarpus-siliculosus for *E. silicolosus* genome, Department of Energy Joint Genome Institute (JGI) for *E. huxleyi* sequences.

*Nannochloropsis gaditana* sequences were retrieved from www.nannochloropsis.org using TBlastN. The multiple sequence alignment (MAS) of protein was performed using ClustalW in BioEdit and muscle (MUltiple Sequence Comparison by Log‐ Expectation). Sequence similarities and secondary structure information were obtained by ESPript, ‘Easy Sequencing in PostScript’, using the crystal structure of spinach major light‐harvesting complex as a reference (1RWT pdb code; Liu *et al*., 2004).

### MS analysis

The green bands visible on the sucrose gradient were harvested with a syringe and then loaded on SDS‐PAGE (precast 12% polyacrylamide SDS gel; C.B.S. Scientific, San Diego, CA, USA). In‐gel tryptic digestion was performed as described in (Shevchenko *et al*., 2006), with the minor modification of using acetonitrile as the organic phase. The MS measurements were performed as described by (Terashima *et al*., 2010) using an Ultimate 3000 nanoflow HPLC system (Dionex, Sunnyvale, CA, USA) coupled with an LTQ Orbitrap XL mass spectrometer (Thermo Finnigan, Waltham, MA, USA) device for autosampling, column switching and nano‐HPLC. For the identification of peptides, omssa (v.2.1.4) (Geer *et al*., 2004). A new database was created by downloading *N. gaditana* sequences from www.nannochloropsis.org/page/ftp to assign the peptides resulting from MS analysis to *N. gaditana* protein.

### Label‐free quantification

For peptide identification and determination of protein intensities, MS raw data were processed using maxquant v.1.5.1.2 (Cox & Mann, 2008). The Uniprot reference proteome of *N. gaditana* (downloaded 12 December 2014) concatenated with randomized sequences of all entries was used for database search, with default settings for high‐resolution MS1 (Orbitrap) and low‐resolution MS2 (ion trap). Oxidation of methionine and acetylation of the protein N‐terminus were allowed as variable modifications. False discovery rate (FDR) was set to 1% at the peptide and protein level. The feature ‘match between runs’ was activated. Due to the fundamentally different protein composition of each fraction, intensity normalization by MaxQuant was omitted. Instead, normalization factors were calculated based on the sums of all non‐normalized protein intensities of each fraction.

### Pigment analysis

Chlorophylls and carotenoids were extracted using 80% acetone, and pigment content was determined by HPLC (Beckman System Gold) as described previously (Färber & Jahns, 1998). The vaucheriaxanthin retention factor was estimated from the one of violaxanthin, with a 10% correction accounting for the different absorption spectrum.

### Spectroscopic steady state measurements

Absorption of isolated PSI‐LHC particles was measured at 77 K and room temperature (RT) with a Varian Cary 4000 UV‐Vis spectrophotometer. For 77 K measurements, samples were in 65% glycerol. Fluorescence spectra were measured at 77 K and RT on a Fluorolog spectrofluorimeter (Jobin Yvon Horiba, Kyoto, Japan). Samples were diluted to Q_y_ optical density (OD_Qy_) 0.07 cm^−1^ in order to avoid self‐absorption in a buffer containing 10 mM Hepes (pH 7.5) and 0.06% α‐DM. The Circular‐Dichroism (CD) spectra were measured using a Chirascan‐Plus CD Spectrometer at RT.

### Time‐resolved measurements – streak camera set‐up

Picosecond‐time‐resolved fluorescence measurements were performed with a streak camera setup as previously described (Gobets *et al*., 2001; van Stokkum & Van Oort, 2008; Le Quiniou *et al*., 2015b). The samples were measured upon 400 nm excitation. The repetition rate was set at 250 kHz, the pulse energy was below 0.4 nJ. The samples were stirred in all cases. A power study confirmed the absence of annihilation in the measuring conditions.

Fluorescence was detected from 590 nm to 860 nm in three different time ranges (TR): 0 to 155 ps (TR1 temporal response 4–5 ps), 0 to 400 ps (TR2 temporal response 6–7 ps) and 0 to 1500 ps (TR4 temporal response 20 ps). The streak camera data were treated in HPD‐TA 8.4.0 (Hamamatsu Photonics, Hamamatsu, Japan) as described in detail in Le Quiniou *et al*. (2015b). The instrument response function (IRF) was modeled with a simple Gaussian for all the TRs. PSI‐LHC particles were diluted at an OD of 0.6 cm^−1^ for PSI‐LHC_α_ and 1.2 cm^−1^ for PSI‐LHC_β_ at the Qy maximum with a buffer containing 10 mM HEPES (pH 7.5) and 0.06% α‐DM. The chosen OD enabled time‐resolved measurements without self‐absorption.

### Data analysis of time‐resolved measurements

The streak camera measurements were analyzed globally in Glotaran (Snellenburg *et al*., 2012) with a sequential model. The average decay time τ_av CS_ (Eqn (Eqn 1)) characterizes the time until charge separation (CS) occurs (for open RCs) with *A*_*n*_ the area under the DAS of the *n* ^th^ component (i.e. its total amplitude) and *A*_*n*_/ ∑ _*n*_ *A*_*n*_ the relative amplitude.

(Eqn 1) $$\tau_{\mathrm{av}\;\mathrm{CS}}=\sum_{n}\left(\tau_{n}\cdot A_{n}\right)/\sum_{n}A_{n}$$

See Le Quiniou *et al*. (2015b) for more details.

## Results

### Structural analysis reveals unusual organization of the peripheral antenna in the PSI‐LHC complexes from *Nannochloropsis gaditana*

**Figure 1:**
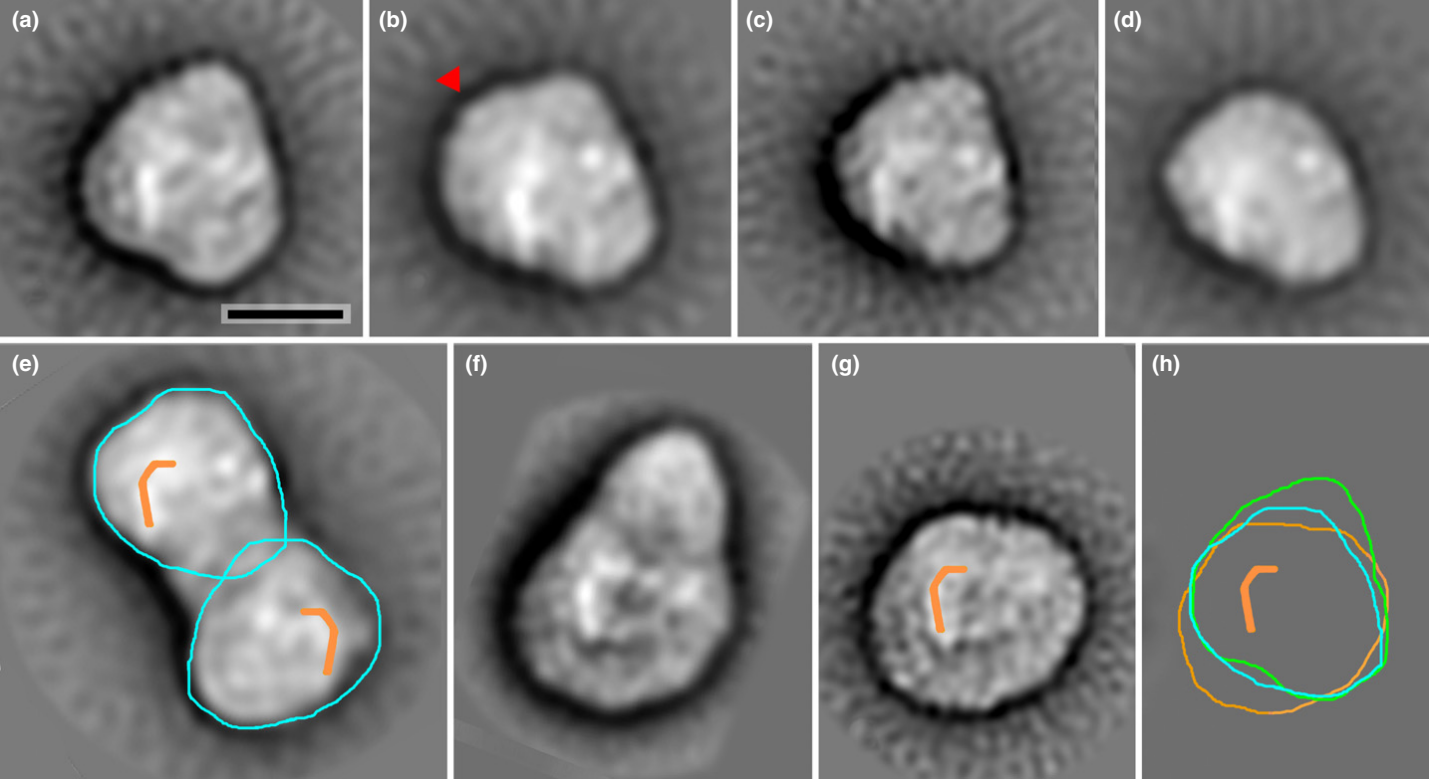
Electron microscopy analysis of Photosystem I light‐harvesting super complex (PSI‐LHC) from *Nannochloropsis gaditana*. (a–d) Projection maps of the *N. gaditana* monomer, sums of four best classes after processing 200 000 particles, see text. The red arrowhead in (b) shows extra density, absent in (a); scale bar, 10 nm. (e) Projection map of the *N. gaditana* dimer, after processing 50 000 projections. (f, g) Maps of *Arabidopsis thaliana* PSI‐LHC and PSI, reanalyzed from Kouril *et al*. (2005b). (h) Aligned contours of *N. gaditana* (a, green, upper monomer of (e) in blue) and *A. thaliana* (g, orange). The ‘reversed J’ motif (orange) and other densities were helpful to align the core parts and also to establish that the dimers of *N. gaditana* (e) consist of up and down oriented monomers, as indicated.

PSI‐LHC can be purified from *N. gaditana* thylakoids in supercomplexes containing the core complex with its associated antenna (Basso *et al*., 2014). The structure of PSI‐LHC from *N. gaditana* purified after solubilization with either α‐ or β‐DM (PSI‐LHC_α_ and PSI‐LHC_β_) was investigated using electron microscopy (EM). Projection maps of negatively stained PSI‐LHC_α_ and PSI‐LHC_β_ were obtained by single particle analysis (Scheres *et al*., 2008; Boekema *et al*., 2009; Scheres & Chen, 2012). In PSI‐LHC_α_ we found two types of particles: a monomeric complex (Fig. 1a–d) and a dimeric complex (Fig. 1e), in a ratio 8 : 2 (*c*. 230 000 and 58 000 particles, respectively). The monomers have a size comparable to that of the PSI‐LHCI isolated from *A. thaliana* (Fig. 1f,g). β‐DM gives harsher solubilization than α‐DM (Basso *et al*., 2014) and PSI‐LHC_β_ samples showed the presence of monomers with < 1% of the particles that could be classified as dimers. An equivalent P700 : Chl ratio was measured in PSI‐LHC_α_ and PSI‐LHC_β_ supporting the hypothesis that monomers and dimers have similar composition (Supporting Information Fig. S1).

In PSI‐LHC_α_ four main types of monomers were found upon classification. The largest ones have a triangular shape (Fig. 1a) and some of them show an extra density on the upper left side (indicted with a red arrow in Fig. 1b). In other particles, this density is less pronounced, which can partly be caused by a tilt of the particles on the carbon support film (Fig. 1c,d). All projection maps show clear densities that in the *A*. *thaliana* map (Fig. 1g) correspond to the extrinsic subunits of PSI core. These densities can then be used for the alignment for *N. gaditana* particles (green contour in Fig. 1h) with that one of *A. thaliana* (orange contour, Fig. 1h). This comparison shows that the *N. gaditana* particles have a different shape than those of *A. thaliana*, being substantially smaller at the bottom but larger at the top, according to the orientation shown in the figure.

In the dimeric complex the upper monomer has exactly the same outline as the monomer of Fig. 1(d), indicating a small loss of density at the tip (blue contour in Fig. 1e,h). The lower monomer of the dimer is, however, not related by two‐fold symmetry to the upper one, as the blue contours plus the orange‐marked density clearly demonstrate. This is only compatible with a flipping plus a rotation, indicating that monomers are oriented upside‐up and upside‐down. Such an opposite orientation of the two monomers suggests that the dimers are artificial, as earlier observed in plants (Kouril *et al*., 2005a).

**Figure 2:**
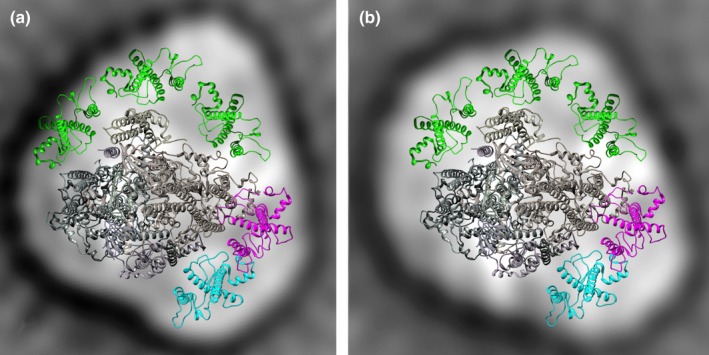
Model for subunit positions in *Nannochloropsis gaditana* Photosystem I (PSI). (a, b) Projection maps of the *N. gaditana* monomer from Fig. 1(a, b) (respectively) overlaid with wire models of the components of the plant high‐resolution protein data bank structure 4XK8 from Qin *et al*. (2015). From the plant model subunits G, H, K and LHCA1/4 were removed and all other subunits were overlaid as a fixed structure and shown in gray. LHCA2/3 are shown in cyan/purple. There is no space left for the two additional light harvesting complexes (LHCs) at the lower left positions next to LHCA2. Instead, additional LHCs (green) have been positioned at the top, to indicate ample space at this site for three extra LHCs, especially (b).

The best two maps of *N. gaditana* (Fig. 2a,b) were overlaid with the atomic model of the plant PSI structure (Ben‐Shem *et al*., 2003; Qin *et al*., 2015). In plants such as pea and *A. thaliana* the LHCA1–4 monomers constitute a belt at one side of the core, whereas in *N. gaditana* there appears to be space only for two LHCs, in a position corresponding to the plant LHCA2/3 dimer, at the lower right tip (Fig. 2a,b). The model further shows space for at least three other LHCs at the other side of the core complex, especially in the more bulky map of Fig. 2(b). This modeling clearly shows that *N. gaditana* PSI has a unique antenna structure, different from plants and green algae, which likely is composed of five LHC subunits associated to two opposite sides of the core complex.

### PSI‐LHC composition in *Nannochloropsis gaditana*

PSI core complex subunits were identified in the *N*. *gaditana* genome using similarity searches with the blastp tool at NCBI (using query sequences from *A*. *thaliana*,* Cyanidioschizon merolae* and *Pheodactylum tricornutum*, Table 1). Sequences of PSI subunits from various additional species from *Viridiplantae* (*A*. *thaliana thaliana*,* O*. *sativa*,* P*. *patens*,* C*. *reinhardtii*), red algae (*G*. *sulphuraria*), heterokonts (diatoms and *E*. *silicosus*) and haptophyta (*E*. *huxleyi*) also were searched to build a comprehensive view of the subunit distribution in different photosynthetic eukaryotes (Tables 1, S1). Some subunits (PsaA‐E), known to be involved in charge separation and electron transport, are highly conserved in all photosynthetic organisms including cyanobacteria. Noteworthy, their conservation is not only limited to the presence/absence of the subunits but also extends to their amino‐acid sequence (Table S2). Other PSI core subunits (PsaF, PsaI, PsaJ and PsaL) are also conserved in all the species analyzed, suggesting that they play a relevant role in PSI structure and function.

**Table 1:** Photosystem I (PSI) core complex subunits

Protein name	Viridiplantae				Rhodophyta		Chromoalveolata				
	Plants (Angio‐sperm)		Plants (Bryo‐phyta)	Green algae (Chloro‐phyta)	Red algae		Heterokontophyta				Hapto‐phyta
	*A. thaliana*	*O. sativa*	*P. patens*	*C. reinhardtii*	*G. sulphuraria*	*C. merolae*	*P. tricornutum*	*T. pseudonana*	*E. siliculosus*	*N. gaditana*	*E. huxleyi*
Present in cyanobacteria											
PsaA	+	+	+	+	+	+	+	+	+	**+**	+
PsaB	+	+	+	+	+	+	+	+	+	**+**	+
PsaC	+	+	+	+	+	+	+	+	+	**+**	+
PsaD	+	+	+	+	+	+	+	+	+	**+**	+
PsaE	+	+	+	+	+	+	+	+	+	**+**	+
PsaF	+	+	+	+	+	+	+	+	+	**+**	+
PsaI	+	+	+	+	+	+	+	+	+	**+**	+
PsaJ	+	+	+	+	+	+	+	+	+	**+**	+
PsaK	+	+	+	+	+	+	nd	nd	nd	**nd**	nd
PsaL	+	+	+	+	+	+	+	+	+	**+**	+
PsaM	nd	nd	+	nd	+	+	+	+	+	**nd**	+
Absent in cyanobacteria											
PsaG	+	+	+	+	nd	nd	nd	nd	nd	**nd**	nd
PsaH	+	+	+	+	nd	nd	nd	nd	nd	**nd**	nd
PsaN	+	+	nd	+	nd	nd	nd	nd	nd	**nd**	nd
PsaO	+	+	+	+	+	+	nd	nd	nd	**nd**	nd

The table shows the identification of PSI core subunits in model species from different taxonomic groups: *Arabidopsis thaliana, Oryza sativa*,* Physcomitrella patens*,* Chlamydomonas reinhardtii*,* Galdieria sulphuraria, Cyanidioschyzon merolae*,* Phaeodactylum tricornutum*,* Thalassiosira pseudonana*,* Ectocarpus silicolosus*,* Nannochloropsis gaditana*,* Emiliania huxleyi*. Presence/absence in cyanobacteria was retrieved from the literature (Jensen *et al*., 2007), whereas for the other species sequences were identified (+) or not (nd) with blastp tool at NCBI (using as query sequences from *A. thaliana* or *C. merolae* and *P. tricornutum* for the subunits absent in *A. thaliana*). The *N. gaditana* column, based on sequences identified in this work, is highlighted. Sequence identification numbers are all reported in Supporting Information Table S1.

Bold highlights the *Nannochloropsis gaditana* that was analysed in this study.

Several other core complex subunits are instead differently distributed among the species analyzed. The *Nannochloropsis gaditana* genome lacks several PSI subunits identified in other organisms, namely PsaG, PsaH, PsaK, PsaM, PsaN and PsaO. As shown in Table 1, PsaG, PsaH and PsaN are present exclusively in some plants and green algae (Khrouchtchova *et al*., 2005). PsaO is instead present only in primary endosymbiotic groups (plants and green/red algae). PsaM, typical of cyanobacteria is also found in some algae species but is missing in others including *N*. *gaditana* (Jensen *et al*., 2007). PsaK showed instead a peculiar distribution because it is conserved from cyanobacteria to red algae and plants but is absent from all heterokonts and haptophytes analyzed, including diatoms and *N*. *gaditana*. These organisms are all secondary endosymbionts originated from a red alga, thus suggesting that PsaK was present in these organisms' ancestors but was later lost.

A similarity search for antenna sequences allowed identification of three subgroups of LHC in *N*. *gaditana*, which were classified as LHCf, LHCr and LHCx (Table 2), as described previously (Vieler *et al*., 2012; Basso *et al*., 2014). Within the three subgroups, proteins were named according to their RNA expression levels, starting from the most actively transcribed (Alboresi *et al*., 2016).

**Table 2:** Light‐harvesting complex (LHC) proteins of *Nannochloropsis gaditana*

Protein name	Gene ID	UniProt ID
LHCf		
NgLHCf1	Naga_100012g50	W7T4V5
NgLHCf2	Naga_100005g99	W7TY83
NgLHCf3	Naga_100157g5	K8Z8N4
NgLHCf4	Naga_100168g14	W7TFG9
NgLHCf5	Naga_100017g59	W7TRI0
NgLHCf6	Naga_100004g86	W7TXE6
NgLHCf7	Naga_100013g28	W7U405
NgLHCf8	Naga_100027g19	W7TCK1
LHCr		
NgLHCr1	Naga_100002g18	K8YRV9
NgLHCr2	Naga_100168g13	W7TZB5
NgLHCr3	Naga_100018g45	K8ZB38
NgLHCr4*	Naga_100056g15	W7TJ16
NgLHCr5*	Naga_100092g17	W7TX20
NgLHCr6*	Naga_100434g4	W7TJ16
NgLHCr7*	Naga_100641g3	W7T8I0
NgLHCr8*	Naga_100017g83	W7UAI7
LHCx		
NgLHCx1	Naga_100173g12	K8YWB4
NgLHCx2	Naga_100056g42	K8YZX9
NgLHCX3	Naga_101036g3	W7TIB0
LHC‐like		
LHC‐like–LIL1*	Naga_100030g5	W7TTN9
LHC‐like–LIL2	Naga_101227g1	W7TI84

LHC are regrouped according to different subgroups as in diatoms (Vieler *et al*., 2012). Sequences of the same subgroup were numbered based on their gene expression levels, starting from the most abundantly transcribed (Alboresi *et al*., 2016). Gene ID is taken from http://www.nannochloropsis.org/ (Corteggiani Carpinelli *et al*., 2014). Proteins (UniProt ID) were identified by mass spectrometry using the UniProt reference proteome of *N. gaditana* (UP000019335) for database search. *Enrichment in Photosystem I supercomplex (PSI‐LHC). The correspondence with proteins from *N. oceanica* is shown in Supporting Information Table S3.

**Figure 3:**
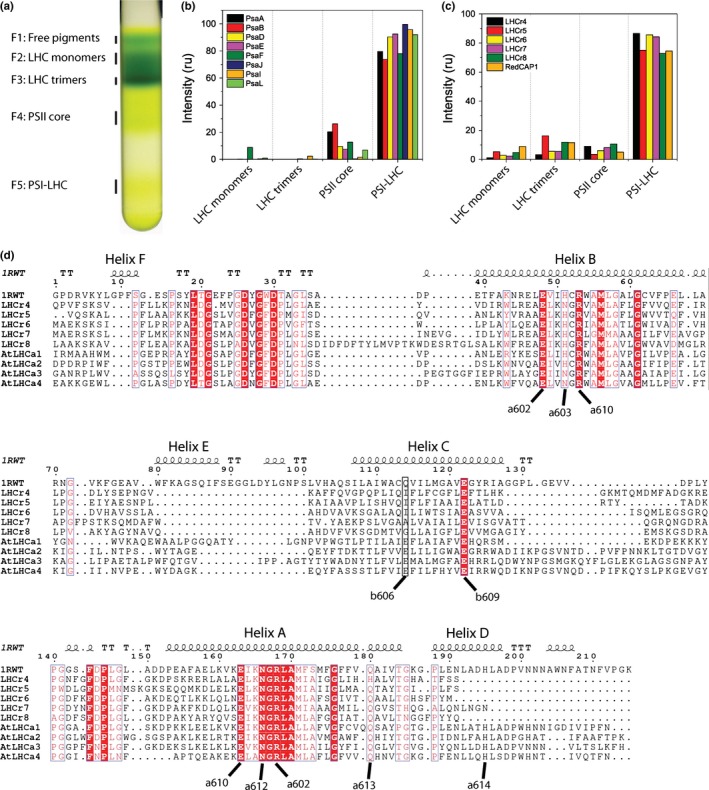
Protein distribution in the different fractions harvested after sucrose gradient sedimentation of solubilized thylakoids. *Nannochloropsis gaditana* thylakoid membranes were first solubilized with α‐DM and then separated by sucrose density gradient ultracentrifugation and subsequently characterized by MS/MS analysis. (a) Example of sucrose density gradient after centrifugation of thylakoid membranes of *N. gaditana*. (b) Distribution of subunits of the Photosystem I (PSI) core complex in the gradient fractions. (c) Light harvesting complexes (LHCs) showing a relative enrichment in PSI fractions, others are shown in Supporting Information Fig. S2. ru, relative units. All *N. gaditana* PSI core subunits and LHCs are listed in Tables 1 and 2. (d) Structure‐based sequence alignment of the crystallized spinach LHCII (Liu *et al*., 2004; pdb code 1RWT) with PSI associated LHCs of *N. gaditana* and *Arabidopsis thaliana*. The secondary structure of the spinach LHCII trimer is shown above the alignment together with the names of the helices. Conserved amino acids highlighted by a red background are identical and those in red letters are similar. Alpha helices are represented as helices. Conserved residues involved in the binding of Chl*a* molecules are labeled under the alignment.

*Nannochloropsis gaditana* thylakoids were solubilized with α‐DM and pigment‐binding protein complexes were first separated in sucrose gradients by centrifugation and then analyzed by tandem mass spectrometry (MS/MS) (Fig. 3). All PSI core and LHC subunits reported in Tables 1 and 2 with the exception of PsaC were detected by mass spectrometry confirming their presence in *N. gaditana* thylakoids. Although it does not provide an absolute quantification, MS analysis allowed determination of the distribution of each polypeptide between the different bands of the sucrose gradient (i.e. LHC monomers, LHC trimers, PSII core and PSI‐LHC). All putative PSI core complex proteins listed in Table 1 showed a strong enrichment (75–100% of the total amount of each peptide) in the PSI‐LHC fraction, confirming that they are indeed components of this supercomplex (Fig. 3b). Five antenna subunits, LHCr4‐8, showed a distribution along the sucrose gradient bands similar to that of PSI core subunits with a strong enrichment in the PSI‐LHC fraction, thus suggesting that they are also specific components of this supercomplex (Fig. 3c). One additional LHC‐like subunit (Naga_100030g5), recently attributed to the group of red lineage chlorophyll binding‐like proteins (RedCAP1) (Litvín *et al*., 2016), was also enriched in the PSI‐LHC fraction. All other LHCs, instead, presented a different distribution and no strong enrichment in PSI fraction (Fig. S2). It is interesting to point out that three other LHCr proteins (LHCr1‐3) were detected in this experiment but not found to be specifically enriched in the PSI‐LHC fraction (Fig. S2). In particular, LHCr1 was present mainly as a monomer whereas LHCr3 was accumulated mainly as a monomer/oligomer (*c*. 80%). LHCr2 showed a significant presence in PSI‐LHC complexes (*c*. 35%) but also in the band of LHC mono/oligomers (*c*. 35%) and PSII core (*c*. 40%). MS analysis thus shows that these subunits are not associated specifically with PSI or at least not as strongly as LHCr4‐8.

Protein sequences of LHCr4‐8 from *N*. *gaditana* were compared with the ones from LHCa1‐4 of *A*. *thaliana* (Fig. 3d). The α‐helices A and B are well conserved including the key residues involved in the binding of Chl *a* molecules (Chl 602, 603, 610, 612 and 613). The α‐helix C is more variable and a clear ligand for Chl 606 is missing, whereas a glutamic acid likely coordinating Chl 609 can be identified. With the exception of LHCr7, α‐helix D is not identifiable in *N*. *gaditana* antennas and thus the ligand for Chl a614 is not conserved as previously observed for LHCb6 of *A*. *thaliana* (Passarini *et al*., 2009). In LHCa complexes Chl 603 is coordinated either to an asparagine or a histidine in the helix B. The presence of an asparagine, as in the case of LHCa3 and LHCa4, was shown to be responsible of a red‐shift in the fluorescence spectrum (Morosinotto *et al*., 2003). LHCr5‐8 have a histidine in that position, whereas LHCr4 has an asparagine suggesting the possible existence of red‐shifted forms in the antenna system of *N*. *gaditana* PSI.

### Functional properties of PSI‐LHC from *Nannochloropsis gaditana*

As expected Chl*a* is the only Chl species present in PSI‐LHC (Table 3). The Chl : Car ratio of PSI‐LHC_α_ is lower than that of PSI‐LHC_β_ suggesting that some of the xanthophylls (mainly violaxanthin) are loosely bound to the complex and lost with the stronger solubilization (Table 3). The Chl : Car ratio of PSI‐LHC is lower (3.2 ± 0.8) than that of *A. thaliana* (4.8 ± 0.1, Wientjes *et al*., 2009) and *C. reinhardtii* PSI (5.0 ± 0.2, Drop *et al*., 2011). When normalized to the same number of Chls, however, the amount of β‐carotene (10.2 ± 2.6 and 12.4 ± 2.1 mol per 100 Chls) is comparable to that of *A. thaliana* (13.1 ± 0.3 mol per 100 Chls), whereas the value of xanthophylls, especially violaxanthin is higher. Because the latter are preferentially bound to the antenna proteins, this suggests a relatively higher carotenoid content in this moiety, in agreement with the low Chl : Car ratio of the LHCs of *N. gaditana* (Basso *et al*., 2014) compared with higher plants (Wientjes *et al*., 2009) and *C. reinhardtii* (Drop *et al*., 2011).

**Table 3:** Pigment content of *Nannochloropsis gaditana* Photosystem I supercomplex (PSI‐LHC)

	Viola‐xanthin	Vaucheria‐xanthin	Antera‐xanthin	Zeaxanthin	β‐carotene	Chl/Car
PSI‐LHC_α_	14.5 ± 2.0	2.3 ± 0.6	2.1 ± 0.8	2.5 ± 2.2	10.2 ± 2.6	3.2 ± 0.8
PSI‐LHC_β_	7.8 ± 2.0	1.1 ± 0.6	< 1	1.2 ± 0.8	12.4 ± 2.1	4.3 ± 0.7

Pigment content of PSI‐LHC fractions purified after different thylakoids solubilization is reported, expressed in mol per 100 Chl. Values are reported as mean ± SD (*n *>* *3).

**Figure 4:**
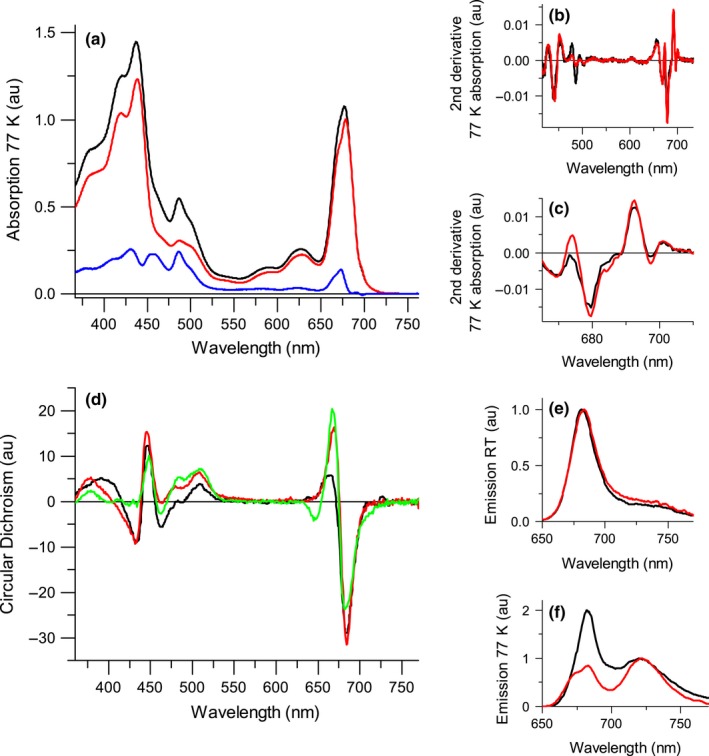
Spectroscopic characterization of the *Nannochloropsis gaditana* Photosystem I supercomplex (PSI‐LHC). (a) Absorption spectra of PSI‐LHC_α_ (black line) and PSI‐LHC_β_ (red line) at 77 K and their difference (blue line). The absorption spectra are normalized to the red absorption from 705 nm and above. (b) Second derivative of the 77 K absorption spectra shown in (a). (c) Enlarged view in the *Q*_y_ region of the 2^nd^ derivative shown in (b). See Supporting Information Fig. S3(a, b) for absorption and second derivatives at room temperature (RT). (d) Circular dichroism (CD) compared with *Arabidopsis thaliana* PSI‐LHCI (green, normalized to the maximum absorption in the *Q*_y_. (e) RT fluorescence emission upon 500 nm excitation, normalized to the maximum emission. See Fig. S3(c, d) for RT emission upon 400 nm. (f) 77 K fluorescence emission upon 400 nm excitation, normalized to the maximum in the red region. au, arbitrary units.

The absorption spectra of the PSI particles at 77 K and RT as well as their second derivatives are presented in Figs. 4, S3 and Table S4. The second derivative of the absorption spectra in the Chl region shows the presence of absorption forms *c*. 669 and 680 nm at RT (Fig. S3; Table S4) and at 669, 679.5, 685 and 697.5 nm at 77 K (Fig. 4b,c; Table S4) for both PSI particles. In the carotenoid region, minima of the second derivative are visible at *c*. 486 nm and *c*. 503 nm at 77 K (Fig. 4b; Table S4). The wavelength of the second minimum and the fact that it is similar in the two preparations suggest that it is the signature of β‐carotene in the PSI core. However, the large difference in the 486 nm signal between PSI‐LHC_α_ and PSI‐LHC_β_ suggests that it originates from xanthophylls (mainly violaxanthin), that are strongly reduced in PSI‐LHC_β_. The Circular Dichroism spectra of the two preparations are shown in Fig. 4(d) together with the spectrum of *A. thaliana*. Although small differences can be observed, the main components of the spectra are present in all complexes indicating that the overall pigment organization is conserved, confirming that the two preparations contain the same complex.

The fluorescence emission spectra at RT have maxima at 681.5 and 683 nm for PSI‐LHC_α_ and PSI‐LHC_β,_ respectively (Fig. 4e). The emission spectra at 77 K (Fig. 4f) of both preparations show an intense band with maximum at 722 nm, typical of the red forms. A second emission band with maximum at 675–680 nm is visible at 77 K, indicating the presence of Chls that do not transfer energy to PSI (see the shift in emission upon 400 nm and 500 nm excitation in Fig. S3c,d).

**Figure 5:**
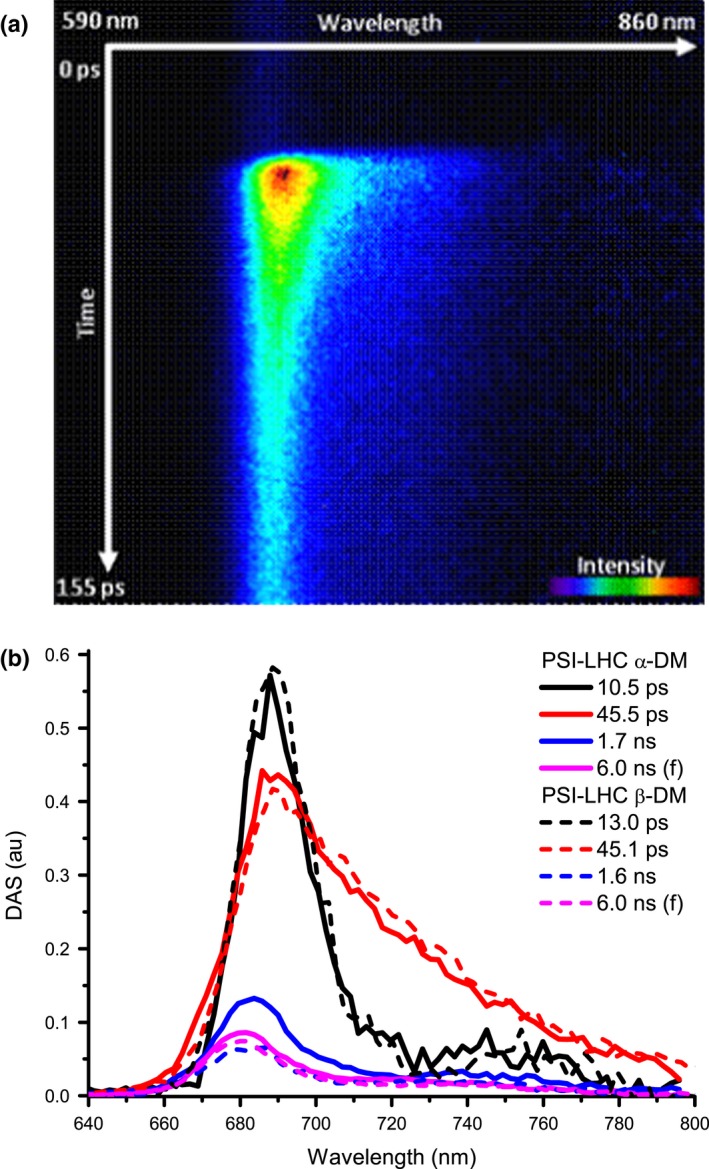
Time‐resolved fluorescence analysis of the Photosystem I supercomplex (PSI‐LHC) for *Nannochloropsis gaditana*. (a) Fluorescence decay of the PSI‐LHC_α_ upon 400 nm measured with the streak camera at the shortest time range (0–160 ps; TR1). (b) Decay‐associated spectra (DAS) of the kinetics components necessary to describe the fluorescence decay from PSI‐LHCI_α_ (solid lines) and PSI‐LHCI_β_ (dashed lines) measured upon 400 nm excitation (normalized to the initial population of excited states in the PSI‐LHC particles).

In order to study excitation energy transfer and trapping, the fluorescence decay kinetics of PSI‐LHC_α_ and PSI‐LHC_β_ were measured with a streak camera set‐up. The fluorescence was imaged along wavelengths (from 640 and 800 nm) and time (for three different time ranges up to 1500 ps). An example of a streak image is shown in Fig. 5(a). After sequential analysis, the global decay of the two samples is described as a sum of decay components (see the Materials and Methods section) and the decay associated spectra (DAS) are shown in Fig. 5(b). A minimum of four components was needed for a good description of the kinetics of both samples. The first component (with a lifetime of 10.5 ps in PSI‐LHC_α_ and 13.0 ps in PSI‐LHC_β_) is mainly a decay component although it still contains some energy transfer features as it can be inferred by the partial absence of the expected positive vibrational band. The second component (with a lifetime of 45.5 ps in PSI‐LHC_α_ and 45.1 ps in PSI‐LHC_β_) is a pure decay component and represents the time when most of the trapping occurs. The two slowest components show blue‐shifted DAS and long lifetimes (1.7–1.6 ns and 6 ns), and correspond to disconnected species. Indeed *c*. 1.7 ns is close to the lifetime of LHCAs of higher plants (Passarini *et al*., 2010; Wientjes *et al*., 2011a). The average decay times are calculated by using Eqn (Eqn 1) (see the Materials and Methods section, Table 4) considering only the components attributed to the PSI‐LHC kinetics (thus 1 and 2) and are 31 ps for PSI‐LHC_α_ and 32 ps for PSI‐LHC_β_ (± 3 ps). The overall trapping time is thus much shorter than that of higher plants (48 ps; Wientjes *et al*., 2011b) or *C. reinhardtii* PSI (50 ps; Le Quiniou *et al*., 2015b).

**Table 4:** Photosystem I supercomplex (PSI‐LHC) fluorescence lifetimes

	PSI‐LHC α‐DM	PSI‐LHC β‐DM
τ1 (ps)	10.5	13.0
Relative amplitude	33.1%	35.5%
τ2 (ps)	45.5	45.1
Relative amplitude	48.9%	52.0%
τ3 (ns)	1.7	1.6
Relative amplitude	11%	6.3%
τ4 (ns)	6.0 (f)	6.0 (f)
Relative amplitude	7%	6.2%
Average decay time τ_av CS_ (ps)	31	32

Lifetimes were obtained from the sequential analysis of the fluorescence decays of PSI‐LHC_α_ and PSI‐LHC_β_ of *Nannochloropsis gaditana* measured upon 400 nm excitation with their relative amplitude (see the Materials and Methods section) and average decay time. The longest lifetime of both samples is attributed to free Chl*a* visible in the steady state emission spectra (Supporting Information Fig. S3c,d) and fixed (f) to 6 ns which corresponds to the free Chl*a* lifetime in acetone (Palacios *et al*., 2002).

## Discussion

### Structural organization and function of PSI**‐**LHC in the heterokont *Nannochloropsis gaditana*

In this work the combination of structural, proteomic and functional analysis provides a comprehensive picture of the structure and composition of Photosytem I light‐harvesting complexes (PSI‐LHC) from *Nannochloropsis gaditana*. EM analysis of PSI purified from *N. gaditana* shows that this is a supercomplex composed of a core complex and a peripheral antenna system, as in all eukaryotes analyzed thus far (Fig. 1). At variance with plants and other algae (Drop *et al*., 2011; Thangaraj *et al*., 2011; Qin *et al*., 2015), however, five LHCs, identified by MS analysis as LHCr4‐8, are found to be associated with *N. gaditana* PSI‐LHC (Table 2; Fig. 3c). MS analysis detected three more LHCr‐type LHCs, eight LHCf, three LHCx, thus covering the entire LHC superfamily identified in the genome of *N. gaditana* (Table 2). These additional LHCs, however, were not specifically enriched in PSI‐LHC suggesting that they are not strongly associated with this supercomplex. It is, however, not possible to exclude the possibility that some additional antenna are loosely associated with PSI *in vivo* but lost during purification.

The superimposition of the *N. gaditana* PSI supercomplex EM map with the high‐resolution structures of plant PSI evidenced a peculiar arrangement of antenna complexes with two LHCs bound in a highly conserved position, the same occupied by LHCa2/3 in plants (Fig. 2). Three additional LHCs are instead found at the other side of the core complex, where PsaL is located.

**Figure 6:**
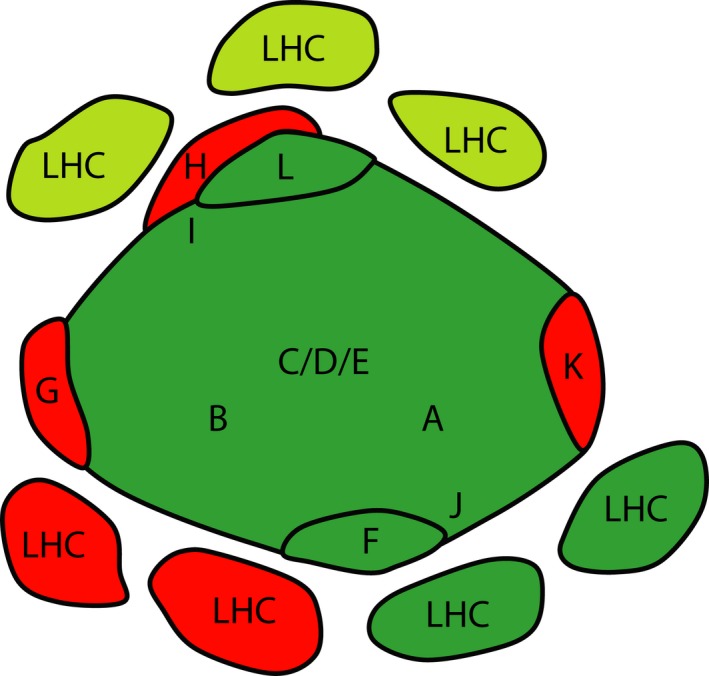
Schematic comparison of Photosystem I supercomplex (PSI‐LHC) structural organization in *Nannochloropsis gaditana* with plants and other algae. Letters from A to K indicate the predicted position of the corresponding Psa subunits. Dark green subunits are those conserved in all eukaryotes (see Tables 1 and 2), red subunits are present in plants, and light green subunits are only in *N. gaditana*.

As schematized in Fig. 6, this peculiar structural organization correlates with differences in the composition of the PSI core complex derived from genome and proteome analysis (Tables 1, 2; Fig. 3). In plants the association of LHCa1/4 with the core complex is partly mediated by PsaG (Ben‐Shem *et al*., 2003; Qin *et al*., 2015). PsaG is absent in *N. gaditana* and indeed no LHC was observed in the position corresponding to plant LHCa1/4. PsaF and J have been suggested to mediate interactions with LHCa2/3 in plants (Qin *et al*., 2015). Consistent with the conservation of PsaF and PsaJ in *N. gaditana*, two LHCs are found in the same position also in this species. Considering that the core complex subunits are well conserved in different organisms (Table 1) this observation also suggests that two LHCs are likely to be bound in this position in PSI from all photosynthetic eukaryotes, including diatoms.

In plants, *psak* knockdown plants showed destabilized LHCa2/3 association (Jensen *et al*., 2000) suggesting that PsaK contributes to their binding to PSI. The recent structure of plant PSI supercomplex, however, showed that PsaK does not interact directly with the peripheral antennae (Mazor *et al*., 2015; Qin *et al*., 2015). PsaK is not found in *N. gaditana* or in any other heterokont analyzed thus far (Table 1) (Le Corguillé *et al*., 2009; Starkenburg *et al*., 2014) likely because of a loss that occurred during or after the secondary endosymbiosis. In these organisms the absence of PsaK does not prevent the association of two LHCs on this side of the core, which confirms that PsaK is not strictly necessary for the association of peripheral antenna.

Additional PSI core subunits identified in plants are not conserved in heterokonts. PsaN in plants is found to be associated with PsaF in the docking site for plastocyanin (Haldrup *et al*., 1999), the soluble PSI electron donor. In *N. gaditana* and other heterokonts only PsaF is present, suggesting that PsaN is dispensable for efficient electron transfer to PSI, consistent with its absence in cyanobacteria. This is likely correlated with a difference in electron transport chain, because *N. gaditana* genome lacks a plastocyanin encoding gene (Corteggiani Carpinelli *et al*., 2014) and the PSI lumenal electron donor is likely cytochrome *c6*, as previously suggested for the red alga *Galdieria sulphuraria* (Vanselow *et al*., 2009) and diatoms (Grouneva *et al*., 2011).

It is also worth underlining the absence of PsaH, a subunit essential for the association of LHCII during state transitions in plants (Lunde *et al*., 2000). In *N. gaditana* other LHCs associate with the core complex in the region generally occupied by PsaH (Fig. 6). This picture suggests that state transitions, if present in *N. gaditana*, most likely involve different structural interactions between antenna and PSI complexes than those described in plants and green algae (Kouril *et al*., 2005b; Drop *et al*., 2014).

The functional data show that, despite this different organization, energy transfer and trapping in the PSI complex of *N. gaditana* is very fast. Indeed, the average decay time of the *N. gaditana* PSI‐LHC monomer is even shorter (32 ± 3 ps) than that of both *Arabidopsis thaliana* and *Chlamydomonas reinhardtii* PSI‐LHCI (*c*. 50 ps; Wientjes *et al*., 2011b; Le Quiniou *et al*., 2015b). This difference can be due to a smaller number of pigments associated with the PSI of *N. gaditana* and/or to a difference in the red forms. It is well documented that the number and the energy of these forms influence the excitation energy migration towards the reaction center in PSI (i.e. more red forms, slower transfer) (Gobets *et al*., 2001; Wientjes *et al*., 2011b; Le Quiniou *et al*., 2015b). Although the number of Chls associated with the LHC in *N. gaditana* is not known and we can thus not exclude that the antenna size of PSI in *N. gaditana* is smaller, it is very likely that part of the observed difference in trapping time is due to the diversity in red forms. Indeed, the red forms of PSI‐LHC of *N. gaditana* are at a higher energy than those of *A. thaliana* as indicated by their emission maximum (722 nm for *N. gaditana* vs 735 nm for *A. thaliana*) and absorption maximum (697 nm in *N. gaditana* vs 705–710 nm in *A. thaliana*; Morosinotto *et al*., 2003; Croce *et al*., 2007) and are then expected to have a smaller influence on the trapping time than the red forms of plants. Independently from the exact origin of this difference, the very fast trapping time observed for PSI‐LHC of *N. gaditana* also indicates that all of the LHC subunits are functionally well connected with the core, allowing for fast excitation energy transfer and high quantum efficiency of energy conversion. This high efficiency is common to all PSI complexes analyzed so far (Wientjes *et al*., 2011b; Le Quiniou *et al*., 2015a,b) and thus appears to be independent of the organization of the antenna around the core because, for example, the position of the additional LHC in *N. gaditana* differs from that of both LHCI and LHCII in plants and *C. reinhardtii* (Kouril *et al*., 2005b; Drop *et al*., 2011, 2014; Qin *et al*., 2015). This suggests that the design of the PSI core allows the functional association of additional subunits to different part of the complex such that even a PSI core completely surrounded by antennae can maintain a very high quantum efficiency.

## Author contributions

T.M. and R.C. planned and designed the research; A.A., C.L.Q., S.Y., M.S., A.M., C.G. and D.S. performed experiments; A.A., C.L.Q., K.N.S.Y., M.S., M.H., E.J.B., R.C. and T.M. analyzed the data; A.A., C.L.Q., S.Y., E.J.B., R.C. and T.M. wrote the manuscript and all authors revised and approved it.

## Supporting information

Please note: Wiley Blackwell are not responsible for the content or functionality of any Supporting Information supplied by the authors. Any queries (other than missing material) should be directed to the *New Phytologist* Central Office.

**Fig. S1** Quantification of P700 content in PSI‐LHC isolated from *Nannochloropsis gaditana*.
**Fig. S2** Protein distribution in the different fractions harvested after sucrose gradient sedimentation.
**Fig. S3** Spectra at room temperature of PSI‐LHC_α_ and PSI‐LHC_β_.
**Table S1** Sequence identification numbers
**Table S2** Identity matrix for PsaA proteins
**Table S3** List of LHC protein orthologs in *Nannochloropsis gaditana* and *N. oceanica*
**Table S4** Spectroscopic data analysis of PSI‐LHC_α_ and PSI‐LHC_β_
