# Supplementary material for: Conservation of core complex subunits shaped the structure and function of photosystem I in the secondary endosymbiont alga Nannochloropsis gaditana
Source: New Phytol. 2016 Sep 13;213(2):714–26. doi: 10.1111/nph.14156 (PMC5216901; doi:10.1111/nph.14156)
Supplement: Supplementary file 1 — Fig. S1 Quantification of P700 content in PSI‐LHC isolated from Nannochloropsis gaditana. Fig. S2 Protein distribution in the different fractions harvested after sucrose gradient sedimentation. Fig. S3 Spectra at room temperature of PSI‐LHCα and PSI‐LHCβ. Table S1 Sequence identification numbers Table S2 Identity matrix for PsaA proteins Table S3 List of LHC protein orthologs in Nannochloropsis gaditana and N. oceanica Table S4 Spectroscopic data analysis of PSI‐LHCα and PSI‐LHCβ [file NPH-213-714-s001.pdf]

Conservation of core complex subunits shaped the structure and function of photosystem I in the secondary endosymbiont alga *Nannochloropsis gaditana*

Alessandro Alboresi, Clotilde Le Quiniou, Sathish K. N. Yadav, Martin Scholz, Andrea Meneghesso, Caterina Gerotto, Diana Simionato, Michael Hippler, Egbert J. Boekema, Roberta Croce and Tomas Morosinotto

Article acceptance date: 13 July 2016

SUPPLEMENTARY MATERIAL

FIGURE S1. Quantification of P700 content in PSI-LHC isolated from *Nannochloropsis gaditana*. The ΔOD_{705} signal measured with a JTS10 spectrophotometer (Biologic, France) was used as a quantification of the total P700 amount present in the sample after normalization to the precise Chl content of each sample (≈ 10 µg of Chl). To obtain the maximal P700 oxidation, P700 of isolated PSI-LHC was fully reduced by incubating the samples in the dark for 5 minutes with 375 µM Methylviologen and 2 mM Ascorbate. After incubation, the sample was illuminated with an actinic red light (630 nm). A) Signal from PSI-LHC$_{\alpha}$ (black squares) and PSI-LHC$_{\beta}$ (red circles) depending on the actinic light intensity (n = 3). B) Average over the different light intensities (150-2050 µmol photons m^{-2} s^{-1}). The average values are not statistically different (n = 12). Error bars represent the standard deviation.

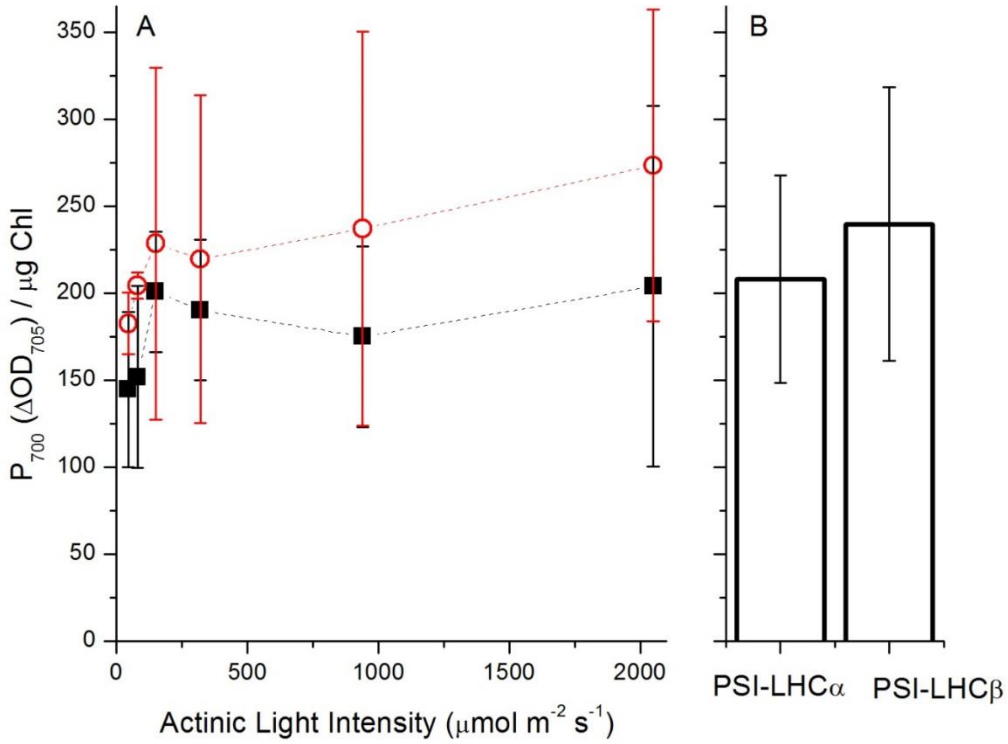

Figure S2. **Protein distribution in the different fractions collected from a sucrose gradient loaded with thylakoids of *N. gaditana* solubilized with α-DM.** Only LHC subunits not specifically enriched in PSI fraction are reported here, divided in LHCF (A), LHCR (B) and LHCX and LHC-like (C). The sucrose density gradient fractions F2-F5 corresponds to LHC monomers, LHC oligomers, PSII core and PSI-LHC, as reported in Figure 3A.

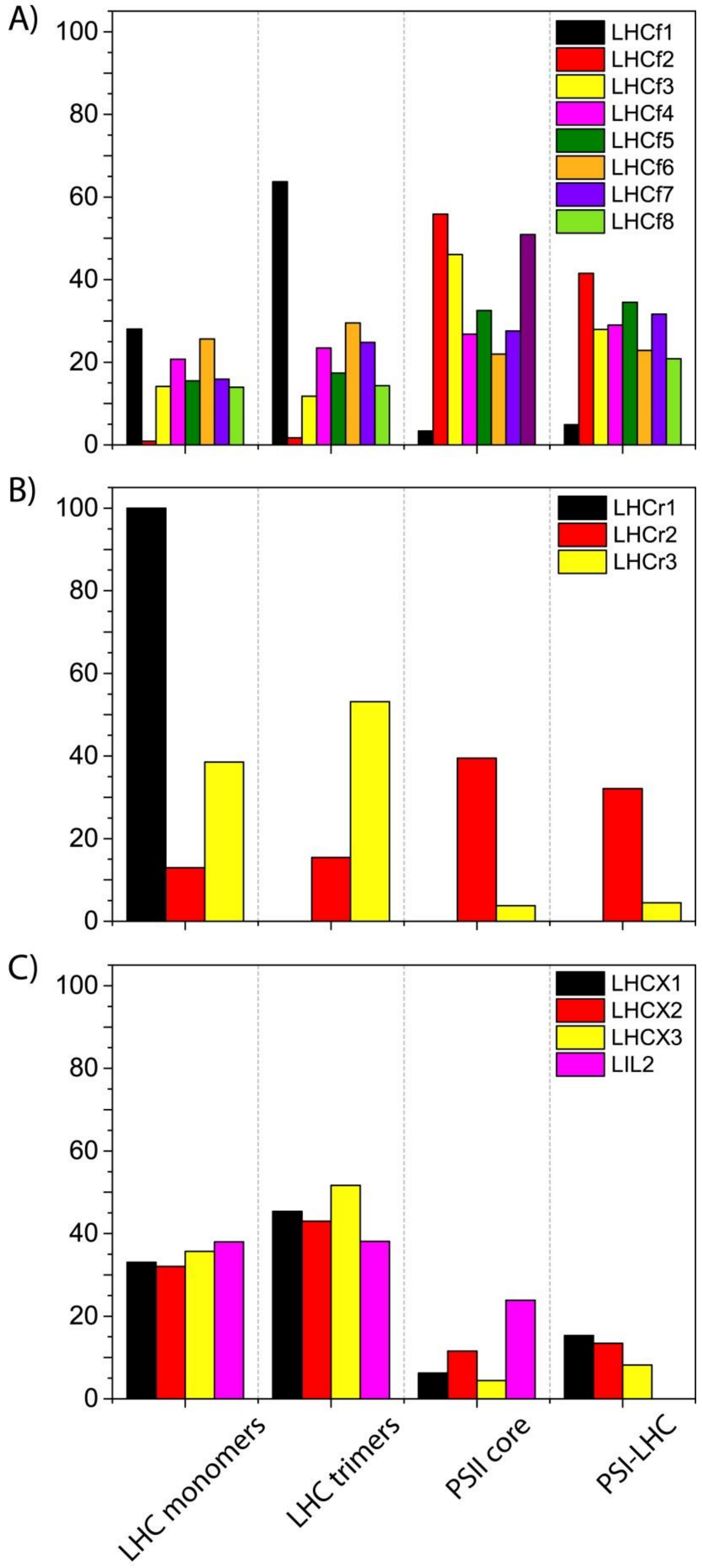

FIGURE S3. Spectroscopic characterization of *N. gaditana* PSI-LHC at room temperature. Absorption spectra (A) of PSI-LHC$_\alpha$ (black) and PSI-LHC$_\beta$ (red), normalized to the maximum in the Q_y region, and their second derivative (B). Fluorescence emission spectra upon 400 nm excitation (narrow line) or 500 nm excitation (thick line) of PSI-LHC$_\alpha$ (C) and PSI-LHC$_\beta$ (D). Spectra are normalized to the maximum emission.

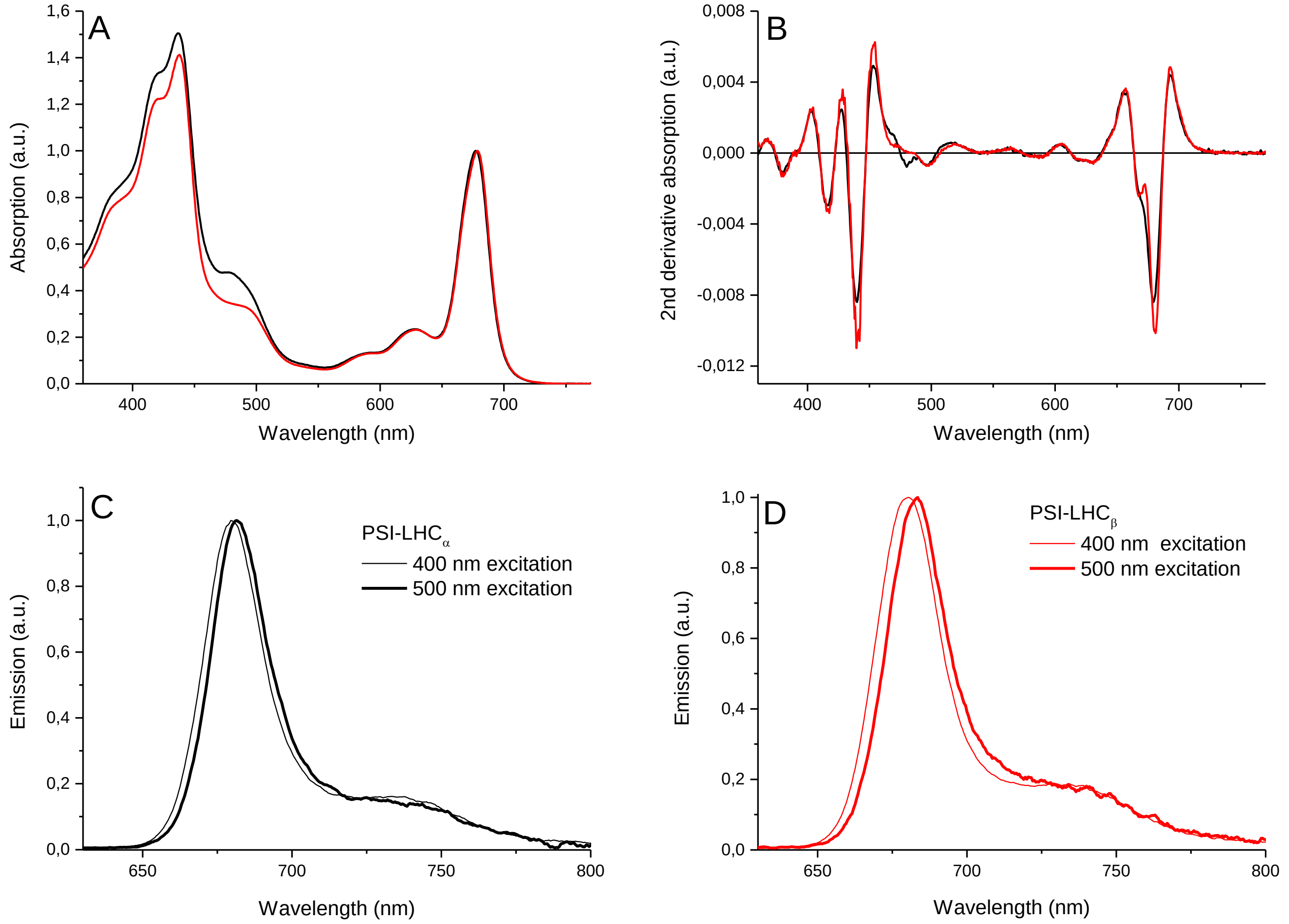

Table S1. NCBI Sequences AC numbers of identified PSI core subunits. * indicates that more than one isoform has been identified in the genome (Jensen *et al.*, 2007; Busch *et al.*, 2013). ND stands for not nedermined sequence.

	subunit	*A. thaliana*	*O. sativa*	*P. patens*	*C. reinhardtii*	*Galdieria sulphuraria*	*Cyanidioschyzon merolae*	*Phaeodactylum tricornutum*	*Thalassiosira pseudonana*	*Ectocarpus siliculosus*	*Nannochloropsis gaditana*	*Emiliania huxleyi*
Present in cyanobacteria	**PsaA**	NP_051059.1	NP_039383.1	NP_904202.1	NP_958375.1	ADO32965.1	NP_849044.1	YP_874359.1	YP_874490.1	YP_003289256.1	YP_007316990.1	YP_277311.1
	**PsaB**	NP_051058.1	NP_039382.1	NP_904203.1	NP_958404.1	XP_005705021.1	NP_849045.1	YP_874358.1	YP_874491.1	YP_003289257.1	YP_007316991.1,	YP_277312.1
	**PsaC**	NP_051110.1	NP_039445.1	NP_904239.1	NP_958423.1	ADO32967.1	NP_848982.1	YP_874487.1	YP_874576.1	YP_003289280.1	YP_007317105.1; AGI98658.1	YP_277346.1
	**PsaD**	NP_192186.1 (D1); *	NP_001062511.1	XP_001751403.1; *	XP_001697722.1	XP_005705023.1	NP_849051.1	YP_874394.1	YP_874547.1	YP_003289238.1	YP_007317065.1	YP_277398.1
	**PsaE**	NP_567818.2 (Ea); *	BAC84088.1	XP_001768221.1; *	XP_001702611.1	ADO32969.2	NP_849039.1	YP_874428.1	XP_002297543.1; *	YP_003289176.1	YP_007317004.1	XP_005784629.1; *
	**PsaF**	NP_174418.1	AAC78106.1	XP_001782922.1; *	XP_001696798.1	XP_005705045.1	NP_849104.1	YP_874361.1	YP_874562.1	YP_003289261.1	YP_007317046.1	YP_277348.1
	**PsaI**	NP_051069.1	NP_039395.1	NP_904192.1	XP_001703367.1	ADO32971.1	NP_849127.1	YP_874378.1	YP_874541.1	YP_003289284.1	AGI98707.1	YP_277394.1
	**PsaJ**	NP_051079.1	NP_039406.1	NP_904180.1	NP_958417.1	ADO32972.1	NP_849105.1	YP_874360.1	YP_874563.1	YP_003289262.1	YP_007317104.1	YP_277349.1
	**PsaK**	NP_174327.1	NP_001058895.1	XP_001752725.1; *	XP_001697230.1	ADO32973.1; P31567.1	NP_848978.1	ND	ND	ND	ND	ND
	**PsaL**	NP_193016.1	NP_001066659.1	XP_001754840.1	XP_001691084.1	ADO32974.1	NP_849135.1	YP_874366.1	YP_874557.1	YP_003289277.1	YP_007317052.1	YP_277352.1
	**PsaM**	ND	ND	NP_904214.1	ND	ADO32975.1	NP_848956.1	YP_874389.1	YP_874533.1	YP_003289254.1	ND	YP_277396.1
Only eukariotic	**PsaG**	NP_175963.1	NP_001063493.1	XP_001765720.1; *	P14224.1	ND	ND	ND	ND	ND	ND	ND
	**PsaH**	NP_188235.1 (H1); *	NP_001056304.1	XP_001766961.1; *	XP_001690629.1	ND	ND	ND	ND	ND	ND	ND
	**PsaN**	AT5G64040	EEC68974.1	Nd	AAK06774.1	ND	ND	ND	ND	ND	ND	ND
	**PsaO**	NP_563815.1	NP_001052755.1	XP_001778960.1; *	XP_001700109.1	XP_005704648.1	XP_005537773.1	ND	ND	ND	ND	ND
	**PsaP**	AT2G46820;	ND	ND	ND	ND	ND	ND	ND	ND	ND	ND

Table S2. Identity matrix for PsaA protein obtained with MUSCLE tool (EMBL-EBI). Conservation versus *A. thaliana* and *N. gaditana* are highlighted in green and yellow, respectively.

PsaA	*Emiliania huxleyi*	*Nannochloropsis gaditana*	*Ectocarpus siliculosus*	*Thalassiosira pseudonana*	*Phaeodactylum tricornutum*	*P. patens*	*A. thaliana*	*O. sativa*	*C. reinhardtii*	*Cyanidioschyzon merolae*	*Galdieria sulphuraria*
Emiliania huxleyi	100	82.95	84.25	85.77	85.11	83.31	80.24	79.71	82.42	82.75	81.52
Nannochloropsis gaditana	82.95	100	85.91	85.5	83.76	82.68	79.6	78.52	82.39	83.06	83.09
Ectocarpus siliculosus	84.25	85.91	100	85.98	85.45	82.44	79.22	78.42	82.89	82.68	81.98
Thalassiosira pseudonana	85.77	85.5	85.98	100	95.48	82.78	79.71	78.5	82.16	81.82	82.31
Phaeodactylum tricornutum	85.11	83.76	85.45	95.48	100	82.11	79.04	77.84	81.62	80.48	81.12
P. patens	83.31	82.68	82.44	82.78	82.11	100	92.67	90.67	89.71	84.89	84.25
A. thaliana	80.24	79.6	79.22	79.71	79.04	92.67	100	95.47	86.36	82.49	81.31
O. sativa	79.71	78.52	78.42	78.5	77.84	90.67	95.47	100	85.03	80.75	80.51
C. reinhardtii	82.42	82.39	82.89	82.16	81.62	89.71	86.36	85.03	100	85.41	84.42
Cyanidioschyzon merolae	82.75	83.06	82.68	81.82	80.48	84.89	82.49	80.75	85.41	100	87.3
Galdieria sulphuraria	81.52	83.09	81.98	82.31	81.12	84.25	81.31	80.51	84.42	87.3	100

Table S3. LHC proteins correspondence between *N. gaditana* and *N. oceanica*. The UniProt ID allowed the identification of the corresponding *N. oceanica* Gene ID. Proposed names of *N. oceanica* CCMP1779 are also reported (Litvín *et al.*, 2016). * indicates proposed attribution to PSI.

	N. gaditana	**Gene ID**	**UniProt ID**	***N. oceanica* CCMP1779**
LHCf	NgLHCf1	Naga_100012g50	W7T4V5	LHCv2
	NgLHCf2	Naga_100005g99	W7TY83	LHCv9
	NgLHCf3	Naga_100157g5	K8Z8N4	LHCv5
	NgLHCf4	Naga_100168g14	W7TFG9	LHCv7
	NgLHCf5	Naga_100017g59	W7TRI0	LHCv4
	NgLHCf6	Naga_100004g86	W7TXE6	LHCv8
	NgLHCf7	Naga_100013g28	W7U405	LHCv3
	NgLHCf8	Naga_100027g19	W7TCK1	LHCv6
LHCr	NgLHCr1	Naga_100002g18	K8YRV9	LHCr5
	NgLHCr2	Naga_100168g13	W7TZB5	LHCr7
	NgLHCr3	Naga_100018g45	K8ZB38	LHCr6
	NgLHCr4*	Naga_100056g15	W7TJ16	LHCr1*
	NgLHCr5*	Naga_100092g17	W7TX20	LHCr3*
	NgLHCr6*	Naga_100434g4	W7TJ16	LHCr4*
	NgLHCr7*	Naga_100641g3	W7T8I0	LHCr2
	NgLHCr8*	Naga_100017g83	W7UAI7	LHCr11
LHCx	NgLHCx1	Naga_100173g12	K8YWB4	LHCx1
	NgLHCx2	Naga_100056g42	K8YZX9	LHCx4
	NgLHCX3	Naga_101036g3	W7TIB0	LHCx3
LHC-like	LHC-like – LIL1*	Naga_100030g5	W7TTN9	Red-CAP1*
	LHC-like – LIL2	Naga_101227g1	W7TI84	LHCv10
				LHCv1
				LHCx2*

Table S4. Maximum absorption of PSI-LHC$_\alpha$ and PSI-LHC$_\beta$ in the Q_y and absorption forms visible in the second derivative of the absorption spectra, at 77K and RT.

	Maximum absorption in the Q_y		2nd Derivative in the Chls region		2nd Derivative in the Car region	
	77K	RT	77K	RT	77K	RT
PSI-LHC$_\alpha$	677.5	677	669, 679.5, 685, 697.5	668.5, 679.5	486.5, 503	480, 497
PSI-LHC$_\beta$	678.5	678.5	669, 679.5, 685, 697.5	667.5, 680.5	486.5, 503	479.5, 497

REFERENCE LIST

Busch A, Petersen J, Webber-Birungi MT, Powikrowska M, Lassen LMM, Naumann-Busch B, Nielsen AZ, Ye J, Boekema EJ, Jensen ON, *et al.* 2013. Composition and structure of photosystem I in the moss Physcomitrella patens. *Journal of experimental botany* **64**: 2689–99.

Jensen PE, Bassi R, Boekema EJ, Dekker JP, Jansson S, Leister D, Robinson C, Scheller HV. **2007**. Structure, function and regulation of plant photosystem I. *Biochimica et biophysica acta* **1767**: 335–52.

Litvín R, Bína D, Herbstová M, Gardian Z. **2016**. Architecture of the light-harvesting apparatus of the eustigmatophyte alga Nannochloropsis oceanica. *Photosynthesis research*. DOI: 10.1007/s11120-016-0234-1.
